# What Do We Talk About When We Talk About Participation? Sense of Community and Social Representations of Participation

**DOI:** 10.5964/ejop.v15i2.1588

**Published:** 2019-06-07

**Authors:** Alessia Rochira, Evelyn De Simone, Terri Mannarini, Sergio Salvatore

**Affiliations:** a Department of History, Society and Human Studies, University of Salento, Lecce, Italy; Webster University Geneva, Geneva, Switzerland; University of Bologna, Bologna, Italy

**Keywords:** sense of community, citizen participation, social representations, analysis of evocations, word association task

## Abstract

The relationship between sense of community (SOC) and citizen participation has been extensively studied in community psychology. Connecting Social Representations and SOC theory, this study explored the lay meanings of citizen participation and its association with SOC. A word association task and a measure of territorial SOC were administered to 390 participants, and data analyzed to explore the contents of the social representations of citizen participation conveyed by the interviewees and their salience. Results revealed that different levels of SOC were associated with variations in the social representation of citizen participation. Specifically, among high-SOC participants the notion of formal political participation prevailed, while among low-SOC participants a more articulated vision emerged, encompassing social and community participation, and also conventional and non conventional types of participatory behaviors.

## Taxonomies of Participation

Citizen participation can be regarded as the active and voluntary involvement of individuals who seek to influence policy decisions and programs, to change problematic conditions in their communities (Ohmer, 2007), modify the material circumstances of their lives and foster well-being (Mannarini, Fedi, & Trippetti, 2009). Researchers from several disciplines, mainly community psychology, sociology, and political science, have contributed to classify various forms of citizen participation. There is a general consensus on the differentiation between *political* and *social* participation. *Political* participation denotes a concern in political life (Rollero, Tartaglia, De Piccoli, & Ceccarini, 2009) and it is conveyed in behaviors such as voting, signing a petition, taking part in a march, belonging to a political party, or running as a candidate. In contrast, *social* participation refers to a variety of pro-social practices beyond the institutionalized political arena, such as volunteering or mobilizing to promote public services, or to defend public spaces. Such a distinction has been further refined with the introduction of the notion of *public* participation (Antonini, Hogg, Mannetti, Barbieri, & Wagoner, 2015), which refers to a socio-political form of participation through which citizens are enabled to express their preferences and concerns about issues of public interest, and to take part in policy-making processes.

Furthermore, Ekman and Amnå (2009), 2012 differentiated *political*, *civil*, and *non- participation* or disengagement. Specifically, *political* participation includes both formal (e.g., voting) and non-formal (e.g., demonstrating) types of citizen participation, with the latter incorporating both legal (e.g., signing a petition) and illegal activities (e.g., sabotage). Additionally, *political* participation encompasses both overt behaviors and cognitive dimensions such as political interest that result in discussing political themes and searching for political information (Almond & Verba, 1963; Marsh & Kaase, 1979). This cognitive participation is often referred to as *latent* participation (Amnå, 2010; Berger, 2009; van Deth, 2001). *Civil* participation consists of committed initiatives towards either specific groups, the entire community, or the broader society. This is the case with recycling, volunteering in the community, taking care of public goods, and being informed about collective issues. Finally, *non-participation* refers to disengagement from both political and social collective issues. Ekman and Amnå (2009) distinguished between active anti-political modes, such as nonvoting, and passive modes, such dismissing any interest or concern in politics.

Besides classifications and taxonomies, meanings of citizen participation in commonsense knowledge have been explored by Social Representations theorists who aimed to explain how the notion and practice of citizen participation were socially constructed by daily communication and interaction to form a shared system of meanings (Jovchelovitch, 2007). Notably, previous studies in the field (Andreouli & Howarth, 2012) posited that citizen participation, and particularly the lay understanding of citizen participation that people produce and share (Mannarini & Fedi, 2010), are sensitive to context, intergroup dynamics, and self-identification with the social setting within which participatory behaviors occur, such as the community or larger social arenas. At the same time, these investigations indicated that social representations of citizen participation may incorporate complementary representations concerning other relevant objects (Andreouli, 2013; Chryssochoou & Lyons, 2011; Howarth, Andreouli, & Kessi, 2014)—for example, the community where the participatory practices occur, the local institutions, the groups or individuals who are perceived as entitled to participate and those who are not—that in turn amplify the social dynamics of inclusion and exclusion. Hence, social representations of citizen participation can be conceptualized as dynamic forms that are located in precise historical and political frames and can vary in their degree of stability and resistance to competing perspectives.

## Citizen Participation and Sense of Community

Sense of community (SOC; Sarason, 1974) is a valuable concept in community psychology that has attracted the interest of hundreds of scholars. According to McMillan and Chavis (1986), SOC encompasses the multifaceted relations between individuals and community. Sense of community includes four key dimensions: the subjective sense of belonging to an organized system (i.e., *membership*), history and collective memories, as well as shared norms, symbols, and experiences (i.e., *shared emotional connection*), the expectation of having personal needs fulfilled by community and its members (i.e., *needs’ fulfillment*), and the sense of both being a source and a target of influence within the community (i.e., *influence*).

Numerous studies have emphasized the positive effects of SOC at both the individual and community levels. In particular, research on participatory behaviors highlighted the positive association between SOC and a diversified range of participatory behaviors has been acknowledged: namely, civic (Brodsky, O’Campo, & Aronson, 1999; Chavis & Wandersman, 1990), social (Obst, Smith, & Zinkiewicz, 2002; Prezza et al., 2001), and political participation (Anderson, 2009), both in conventional and non-conventional forms (Davidson & Cotter, 1986; Xu, Perkins, & Chow, 2010). A recent meta-analytic review (Talò, Mannarini, & Rochira, 2014) attested the existence of a moderate relationship between SOC and citizen participation, especially in cultures where civic and political engagement are valued. In contrast, qualitative analyses found different patterns of relationship: for instance, Mannarini and Fedi (2009) suggested that even a weak SOC can be associated with active citizen participation, as feelings of detachment from the community can enable a more critical and thorough comprehension of its problems, thus encouraging individuals to undertake social and political action. They also pointed out that variations in SOC were associated with different representations of the community (Mannarini & Rochira, 2014), which, in turn, activated distinct forms of citizen participation. These findings suggest the need to considering not only the magnitude and direction of the association between SOC and citizen participation, but also the meanings attributed to both notions in the personal experience of individuals. In particular, our study was built to empirically explore the possibility that differences in individual sense of belonging to the community (i.e., SOC) might be associated with different representations of the concept of citizen participation (Rochira, Fasanelli, & Liguori, 2015). A theoretical and empirical route to delve in such an issue is offered by the Theory of Social Representations and in particular by the structural approach to the study of social representations (Abric, 2001).

## The Theory of Social Representations

In general, the Theory of Social Representations (hereafter TSR; Moscovici, 1973, 1984, 2005) concerns both the process of formation of the social knowledge and its products. With concern to the former, social representations (hereafter SRs) refer to the process by which people create relatively enduring views of important objects and significant events that constitute their reality. With concern to the latter, SRs indicate the structures of knowledge that enable individuals to “classify persons and objects, to compare and explain behaviors and to objectify them as parts of [their] social setting” (Moscovici, 1988, p. 214). Operatively, SRs allow individuals to transform an object, which is perceived as unusual, into something that sounds familiar through associations to those images, concepts and languages—that together form a social representation—that are already known, explicitly through the process of familiarization (Markovà, 2000).

The structural approach to the study of social representations (Abric, 2001) and particularly the Theory of Central Nucleus (TNC), conceptualizes SRs as “organized systems, [that] have two components: content and structure” (Abric, 2003, p. 59). *Content* stands for the information that a group or community share about an object of knowledge, and *structure* refers to the organization of content into a coherent form. According to the TNC, the structure of the representation consists of two main sections: the central *nucleus* and the *periphery*. The nucleus incorporates the most stable and shared information; it also regulates the organization of all the elements of the representation, thereby determining the global meaning of the representation. In contrast, the *periphery* is the most changeable part of the representation that filters the social circumstances and interindividual differences among community or group members (Flament, 1989). The varying configurations of the peripheral elements support the core meaning conveyed by the nucleus, yet they integrate it with individuals’ stories, experiences, and subjective standpoints.

## The Study

The present investigation was part of a larger study aimed at exploring the impact of the financial crisis that has been occurring in Europe in the last decade, on the social identities of European citizens. The project, named Re.Cri.Re—*Between the Representations of the Crisis and the Crisis of Representations*—received a grant within the Horizon 2020 program^i^.

In light of previous studies revealing that variations in SOC might be associated with different forms of citizen participation, the present study aimed to explore to what extent different levels of SOC might be related to different social representations of citizen participation. Specifically, within the TNC framework, the aim was to identify differences between both the contents and the internal structure of the representations of citizen participation according to sub-groups of community members with different levels (i.e., low, medium and high) of SOC.

In line with SRT tenets, our main hypothesis was that members of a local community would share the same set of references, and that this set would characterize the nucleus, but that differences between high, medium and low SOC subgroups would be found on the periphery.

Our secondary hypothesis was that, based on the strand of research attesting a positive association between SOC and citizen participation, residents with a stronger attachment to their community (high-SOC subgroup) would share a more positive image of citizen participation compared to fellow citizens with a weaker attachment (medium and low SOC subgroups).

Finally, our research intended to detect the contents of the lay representations of citizen participation produced by community members, and to compare them to the theoretical definitions and typologies available in the literature.

## Method

### Participants

A convenience non-proportional quota sample by age and gender of residents in the Salento territorial community (Southern Italy) was selected. The sample was composed of 390 participants (49% female), all native-born Italian, aged between 19 and 94 years (*M* = 42.48, *SD* = 14.66). Subjects who not accomplished the word association task were excluded and valid cases retained for the analyses amounted to 376. Almost all participants were highly educated. In fact, 47.4% were high school graduates, 30% were college graduates, and 13.6% had post-graduate education. The remaining 9% had lower education levels. 31, 8% of the interviewees identified themselves as left-oriented, 13.3% were right-and 12.1% as center-oriented. A significant portion of the participants (42.3%) declared that they had no political preference.

### Instruments

Data were collected through a self-administered questionnaire. For the purpose of the present study, the following measures were adopted:

#### Word Association Task

The word association task was intended to capture the semantic contents of the social representations, in line with research that studies social representations through the analysis of the semantic elements and their reciprocal relations (Bauer & Gaskell, 2008; Lahlou & Abric, 2011). Participants were presented the stimulus “Participation,” and asked first to freely list the first five words—whether nouns, adjectives, verbs, etc.—that came to their minds, and, second, to rank them by importance (i.e., 1 = most important).

#### Brief Sense of Community Scale

The 8-item version of the Sense of Community Scale (BSCS-8 by Peterson, Speer, & McMillan, 2008) was used to measure SOC with reference to participants’ community of residence. Examples of items are “*I can get what I need in this community*” (i.e., needs fulfillment), “*I feel like a member of this community*” (i.e., membership), “*I have a say about what goes on in my community*” (i.e., influence), and “*I feel connected to this community*” (i.e., shared emotional connection).

#### Socio-Demographics

Participants were asked to provide demographic information specifying their age, gender, level of education, and professional position. Political orientation was assed whereby a single self-report item.

### Analyses

BSCS-8 was checked for reliability, and the value obtained was satisfactory (Cronbach’s alpha = .81). Data collected through the word association task was revised to correct type errors and reduce ambiguities. In particular, given that the software used for data analysis differentiated central and peripheral elements of the social representation computing the frequency of the words evocations, we edited ambiguities to avoid data dispersion. Precisely, compound nouns were joined together to form a single word—for instance the words local and governance, which were written on the same line, were joined together whereby an underscore to form a compound word; feminine was reversed to masculine, and plural forms were converted to singular forms. Moreover, homographs were disambiguated according to participants’ memos—for instance the Italian word *legge* which means both the present third-person singular form of the verb to read (i.e., *legge_verb*) and the noun law (i.e., *legge_noun*).

Participants were divided into the three subgroups according to their SOC score, precisely below the 33° percentile (low SOC), between the 33° and the 66° percentile (medium SOC) and above the 66° percentile (high SOC). The low SOC group comprised 132 participants (SOC, *M* = 16.66; *SD* = 2.59), the medium SOC group included 149 residents (SOC, *M* = 21.49; *SD* = 1.12) and the high SOC group gathered 95 interviewees (SOC, *M* = 26.15; *SD* = 2. 4). Words associations were analyzed separately for the three subgroups.

The data analysis process consisted of two basic steps and was performed separately for each dataset.

First, the semantic data was submitted to a categorization. An open coding was performed to categorize synonyms and capture convergent contents (Strauss & Corbin, 1990). Pairs within the research team coded the sematic data separately for each dataset, compared their coding, and discussed any divergences in coding between them.

Second, each dataset was processed by the software *Ensemble de Programmes permettant L'Analyse des Évocations* EVOC, 2005 (Vergès, Scano, & Junique, 2002). The software EVOC differentiated the semantic elements of the central nucleus and the periphery of the social representation based on the frequency hierarchy and average order of the evocations (i.e., rank)—along with the Zipf’s law of word frequency distribution (Zipf, 1949). In particular, the Zipf’ law posits that natural language follows a systematic frequency distribution according to which there are very few high-frequency words and many low-frequency words (Piantadosi, 2014).

Precisely, following the approach of EVOC as originally developed Pierre Vergés (Vergés, 1994, 2002), the central nucleus of the social representation was characterized by the words with the highest frequency of occurrence and the lowest rank of appearance—that is, the words that were rated by the participants as the most important. They were the most frequently mentioned terms that provided meaning and stability to the representation. The elements characterized by a high frequency and a rank of appearance above the average level featured the high periphery of the representation. These components were considered part of the first periphery, which can migrate to the central nucleus. The components with average frequency of appearance and low rank were conflicting elements characterizing the contrast zone of the representation. These terms could either typify the nucleus and the first periphery or symbolize the tendencies of a minority. Finally, the elements characterized by low frequency and the highest rank of appearance—that is, the words rated by the interviewees as the least important—were more clearly peripheral elements and belonged to the second periphery.

**Figure 1:**
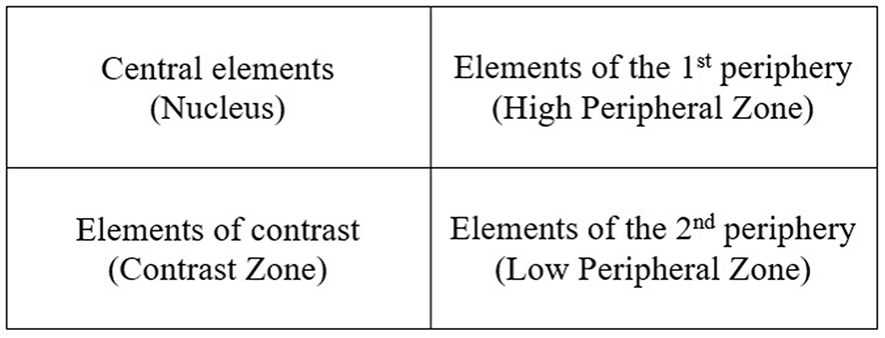
Internal structure of social representations.

Accordingly, the internal structure of the social representations shared by each subgroup resulted in four quadrants (Figure 1) where the central nucleus was situated in the upper left quadrant, the 1^st^ periphery in the upper right quadrant, the contrast zone in the lower left quadrant and the 2^nd^ periphery was located in the lower right quadrant.

Finally, for the purpose of the results description, Italian words were translated into English using back-translation with an English mother tongue speaker for checking the translation accuracy of the word association task data.

## Results

### High-SOC Subgroup

Table 1 displays the internal structure of the social representations of citizen participation within the high-SOC subgroup.

**Table 1:** The Internal Structure of the Social Representation of Participation Produced by High SOC Group

Nucleus			First Periphery		
Word	Frequency ≥ 7	Rank ≤ 3	Word	Frequency ≥ 7	Rank > 3
Election	22	2.773	Manifestation	11	3.455
Collaboration	14	2.714	Responsibility	10	3.200
Duty	10	2.600	Engagement	10	3.400
Union	10	2.600	Agency	8	3.250
Freedom	10	2.700	Politics	8	3.250
Activism	9	2.444	Civicness	7	3.571
Democracy	9	1.778	Sharing	7	3.714
Rights	9	2.889			
Information	9	3.000			
Development	7	2.571			
Fundamental	7	2.286			
Job	7	2.857			
Equality	7	2.571			
Contrast Zone			Second Periphery		
Word	Frequency < 7	Rank ≤ 3	Word	Frequency < 7	Rank > 3
Fight	6	2.500	Membership	5	3.400
Scarce	6	2.667	Association	5	3.200
Involvement	6	2.500	Change	4	3.250
Solidarity	5	2.800	Length	4	3.500
Ineffective	5	2.400	Discontinuity	5	3.200
Interest	5	1.400	Debate	5	4.000
Lawfulness	5	2.200	Ignored	4	3.250
People	4	2.750	Honesty	4	3.250
Referendum	4	2.250	Opinion	5	3.400
Volunteering	4	2.750	Revolution	4	3.250
Awareness	4	3.000	Value	4	3.500
Disagreement	4	3.000	Will	4	3.750

In general, the frequency values of the semantic categories occurring in the nucleus and the 1st periphery of the representation suggested that there was not a clear-cut differentiation between these two areas. At the same time, the value of the rank of appearance indicated that the core of the representation revolved around two basic themes. On the one side, the formal dimension of political participation echoed by the terms *election*, *democracy*, and *rights*; on the other side, also words alluding to commitment and social companionship such as *collaboration*, *union*, and *equality* hinted at the communal side of participation, whereas term *activism* addressed aspects related to agency.

One more significant dyad of words—namely, *development* and *fundamental*—captured a positive view of citizen participation and its prospective outcome.

The 1^st^ periphery of the social representation shared by high-SOC participants was partly aligned with the nucleus, but it included semantic components that point to the unconventional forms of citizen participation. In fact, on the one hand, *politics* reiterated the notion of formal political participation, on the other hand, *manifestation* and *civicness* clearly belonged to social forms of collective action and civic participation. Further elements, such as *responsibility*, *engagement*, *agency*, and *sharing* consolidated the agentic role of social actors and the idea of social commitment.

The contents of both the contrast zone and the 2nd periphery were in part consistent with the nucleus and the 1^st^ periphery of the representation. In particular, several references resonated the formal and civic facets of participation—such as, respectively, *lawfulness*, *referendum* and *volunteering* together with *solidarity*, and *association*. However, a few semantic traits included in the two sections challenged the positive and highly consensual view of citizen participation emerged from the central nucleus. In fact, both conflict and criticism emerged—as shown by the terms *fight*, *scarce*, *ineffective*, *discontinuity*, and *revolution*. These components seemed to convey a complaint concerning the practice of citizen participation.

### Medium-SOC Subgroup

Words occurring in the nucleus were more numerous than those occurring in the other areas of the representation as displayed by Table 2.

**Table 2:** The Internal Structure of the Social Representation of Participation Produced by Medium SOC Group

Nucleus			First Periphery		
Word	Frequency ≥ 6	Rank ≤ 3	Word	Frequency ≥ 6	Rank > 3
Election	32	2.594	Volunteering	13	3.308
Rights	19	1.947	Civicness	12	3.250
Information	19	2.368	Interest	12	3.167
Manifestation	17	3.000	Sharing	12	3.333
Involvement	16	2.188	Politics	12	3.667
Democracy	16	2.438	Association	11	3.455
Freedom	15	2.000	Decision	9	4.111
Referendum	15	2.867	Respect	7	3.429
Engagement	15	2.933	Discontinuity	7	3.571
Responsibility	15	2.800	Deliberation	7	4.429
Collaboration	14	2.929	Agency	6	3.167
Scarce	14	2.929	Organization	6	3.333
Duty	13	2.615			
Activism	13	2.923			
Honesty	10	2.200			
Union	10	3.000			
Will	9	2.778			
Fundamental	8	1.625			
Equality	8	2.750			
Dialogue	6	2.833			
Development	6	2.833			
Voice	6	1.833			
Community	6	3.000			
Contrast Zone			Second Periphery		
Word	Frequency < 6	Rank ≤ 3	Word	Frequency < 6	Rank > 3
Communion	5	1.800	Disinterest	4	3.250
Culture	4	2.000	Justice	4	3.250
Relations	5	2.000	Political Party	4	3.250
Fight	5	2.000	People	4	3.250
Expression	5	2.400	Apathy	4	3.500
Representative Democracy	5	2.400	Opinion	4	3.500
Need	4	2.500	Assembly	5	3.600
Hope	4	2.500	Awareness	5	3.600
Collectivity	5	2.600	Internet	4	3.750
Ineffective	4	2.750	Job	4	3.750
Opportunism	4	2.750	Utopia	5	3.800
Government	4	3.000	Realism	4	4.250
Debate	4	3.000	Confusion	4	4.750
			Positive Emotions	5	4.200
			Dissatisfaction	4	4.250
			Revolution	4	4.750
			Choice	4	4.000

Such a distribution suggested that the kernel of the image of citizen participation shared by the medium SOC subgroup was varied in content and highly consensual, with moderate divergences in the peripheral areas.

Indeed, the core of the representation conglobed references to the political and institutional as well as the social participation. In fact, on the one hand, the lemmas *election*, *rights*, *democracy*, and *referendum* delineated the institutional mode of participation. On the other hand, *manifestation*, *collaboration*, *union* and *community* mainly designated the social counterpart of participation together with its civic mode evoked by the semantic traits *volunteering*, *civicnes*, *association* and *agency*, that characterize the 1^st^ periphery.

Individual agency and responsibility confirmed to be central elements, as indicated by the words *involvement*, *engagement*, *responsibility activism* and *will*—included in the nucleus—and *interest decision* and *agency*—comprised in the 1^st^ periphery. At the same time, these areas encompassed the dialogic facet of citizen participation outlined by the terms *dialogue*, *voice*, and *deliberation*; though, the frequency and rank values informed that this semantic component is less important than, respectively, the political/institutional view and the social view of citizen participation.

The elements contained in both the contrast zone and the 2^nd^ periphery aligned with the semantic contents characterizing the core and 1^st^ periphery of the representation, although a few original constituents emerged. In particular, whereas the contrast zone collected abstract building blocks evoking the subjective and relational texture of citizen participation—for example, *communion*, *relations*, *culture*, *need* and *hope*—the 2^nd^ periphery introduced references to everyday life experiences, such as *job* and *internet*. Additionally, although marginally, conflicting and unconventional participatory practices were mentioned both in the contrast and in the 2^nd^ periphery—see *fight* and *revolution*.

### Low-SOC Subgroup

The examination of the internal structure of the social representation shared by the low-SOC subgroup of participants (Table 3) showed that the core, that is the one conveyed by the nucleus and the 1^st^ periphery, was characterized by a bottom-up view, as indicated by the references to *activism*, *collaboration*, *people*, *common goods*, *union*, *community* and *society*. Moreover, the core transmitted the values underlying the representation of citizen participation, such as *freedom*, *equality*, and *honesty*. However, the kernel of the shared image of participation also included the institutional mode that is reminded by the semantic component *rights*.

**Table 3:** The Internal Structure of the Social Representation of Participation Produced by Low SOC Group

Nucleus			First Periphery		
Word	Frequency ≥ 6	Rank ≤ 3	Word	Frequency ≥ 6	Rank > 3
Freedom	19	2.053	Election	28	3.107
Activism	17	3.000	Sharing	15	3.200
Rights	17	2.588	Manifestation	13	3.308
Information	15	1.667	Referendum	11	3.091
Equality	12	2.333	Scarce	10	3.200
Democracy	12	2.500	Ineffective	10	3.900
Responsibility	10	2.300	Volunteering	10	3.900
Collaboration	10	3.000	Union	9	3.222
Awareness	8	3.000	Politics	9	3.556
People	7	2.429	Agency	8	3.125
Deliberation	6	2.167	Mendacity	8	3.500
Apathy	6	1.333	Interest	7	3.429
Common Goods	6	2.333	Transparency	7	3.714
Expression	6	2.333	Choice	7	3.571
Honesty	6	3.000	Selfishness	6	3.667
			Involvement	6	3.333
			Community	6	3.333
			Society	6	3.500
Contrast Zone			Second Periphery		
Word	Frequency < 6	Rank ≤ 3	Word	Frequency < 6	Rank > 3
Membership	5	2.000	Hear	4	3.250
Confusion	4	2.750	Assembly	5	3.800
Constitution	4	2.500	Lacking	5	4.200
Disinterest	5	2.800	Association	4	3.500
Duty	4	2.000	Civicness	4	3.500
Fundamental	4	1.750	Control	5	3.200
Human Being	4	2.250	Culture	4	3.750
Ideas	4	2.750	Decision	5	3.800
Education	4	2.500	Dialogue	4	4.000
Motivation	4	1.750	Disagreement	5	3.200
Opinion	4	3.000	Development	4	4.250
Pluralism	5	2.600	Guarantee	4	3.500
Relations	5	2.800	Governance	4	3.250
			Local Governance	5	3.200
			Engagement	5	3.400
			Useless	4	3.500
			Political Party	5	3.600

The most significant semantic pieces of the 1^st^ periphery established the formal and political mode of participation, as epitomized by the elements *election*, *referendum*, and *politics*. Precisely, the frequency and rank values of *election* indicated that this component can be included in the kernel of the representation.

At the same time, this area comprises allusions to the social—*manifestation*—and civic—*volunteering*—forms of citizen participation. Interestingly, a cluster of semantic categories outlined individual agency and responsibility, that is the subjective capacity to act independently and make free choices, in particular *responsibility* and *awareness*—in the central nucleus—and *agency*, *interest*, and *choice* in the 1^st^ periphery.

The content that characterized the contrast zone and the 2^nd^ periphery was composed of many different elements. Although the values of frequency and rank were modest, they are worth illustrating.

In addition to mentions to the institutional—for example, *constitution*—and civic participation—for example, *civicness*, *assembly* and *association*—the semantic components included in these areas—for example, *ideas*, *education*, *opinion*, *pluralism* together with *disagreement* and *dialogue*—introduced the notion of citizen participation as a cultural and dialogical practice.

Finally, the representational contents revealed ambivalent attitudes. In fact, while a positive evaluation—evoked by the word *fundamental*—distinguishes the whole structure of the representation, criticisms and negative assessments characterized the 1^st^ periphery and the contrast zone; moreover, the more the 2^nd^ periphery is approached, the more the view of citizen participation appeared conflicting, as indicated by the terms *selfishness*, *confusion*, *disinterest*, and *useless*.

### High, Medium and Low SOC Groups Comparison

In the three conditions (e.g., low, medium and high SOC) the structure of the representation revealed a conspicuous set of shared core meanings—as indicated by the number of elements comprised in the central nucleus of the representations. Moreover, noteworthy commonalities and differences in the contents of the social representations of citizen participation shared by the three subgroups of interviewees emerged.

Interestingly, *information* appeared as a significant aspect of the nucleus of the representation of the three groups, evoking the latent component of citizen participation, which relies on individuals’ interest in public issues. References to *political participation*—mainly in its formal variant, that is voting (e.g., election)—were stable across the groups. However, whereas in the high SOC condition the institutional and political view of citizen participation came about as the most important, in the medium SOC it was complemented by the social mode and in the low SOC any particular form prevailed but the bottom-up participation.

Specific of the high SOC subgroup representation were the emphasis on the institutional foundation of citizen participation, the concept of participation as a duty and responsibility, the reference to the issue of job, mentions to development and progress as outcomes of citizen participation, and an overall idealistic view and positive attitude towards it. Unlike the low SOC subgroup, a very few criticisms and bottom-up participation characterized the periphery of the representation.

The contents of the medium SOC representation are scattered across the four sections. Specific to this group were acknowledgments to the personal commitment and free engagement towards citizen participation, the dialogic tenet of citizen participation, references to the civic mode which characterized the 1^st^ periphery of the representation, mentions to culture and intangible aspirations as well as a critical viewpoint which covered the four sections.

Finally, specific of the low SOC subgroup representation were mentions of social/civic participation, along with the political form. References to the social, interactive and communal basis of citizen participation elicited the bottom-up approach, and a critical/disapproving attitude towards individual apathy and selfishness. In addition, likewise the medium SOC subgroup, remarks to education, relations, pluralism and ideas were peculiar to this representation.

## Discussion

As expected in our main hypothesis, participants converged toward a common set of representational elements underpinning the lay notion of citizen participation, regardless of the degree of identification and attachment to the local community. Though, differently from our hypothesis, the intensity of the sense of community was also associated with variances in the core of the representations and not only in its peripheral areas.

There was a large consensus among the participants about the values which constitute the social reality of citizen participation. At the same time, the latent dimension of citizen participation (Amnå, 2010; Berger, 2009; van Deth, 2001), considered as a psychological and pre-political mode of participation (Almond & Verba, 1963; Marsh & Kaase, 1979), also surfaced as a core component of the social representations elaborated by our participants.

The core meaning of the representations conveyed by the three subgroups recalled the definitions, common in the scientific literature, of political, civic and social participation. The emerging consensual reality not related to a general agreement or the same view of citizen participation among the three subgroups. In fact, voting was the participatory behavior largely mentioned by participants. However, the formal and institutional type of political participation was especially evoked in the high SOC subgroup whereas volunteering, demonstrating, or taking part in a community group or meeting were more peculiar of the medium and the low SOC.

Results also revealed that in the specific commonsensical view of citizen participation analyzed in this study, different types of participation were set in different types hierarchy of representativeness with the political mode perceived as the most prototypical when participants having the greater attachment towards the community and the bottom up participation was exemplary to those who were less connected to their community. Broadly, the findings indicated that either a solid or a feeble attachment relationship with the community was related with a preference towards a precise mode of citizen participation. Diversely, an intermediate level of SOC not privileged a well-defined formula of citizen participation but both the political and the social/civic ways intersected between each other’s. These results contended with the idea that different levels of SOC can be associated with different views of citizen participation (Mannarini & Fedi, 2009)

Overall, these findings highlighted that that the lay notion of citizen participation that was detected in our study covered few behavioral habits compared to the repertoire of multiple activities and actions considered in the scientific taxonomies. At the same time, few original contents also emerged—such as dialogue, culture and education—that have not been extensively addressed in such taxonomies, thus suggesting the need for refining or broadening the typologies of citizen participation.

As also supposed in our main hypothesis, differences were found in the peripheral part of the representations, according to different level of individuals’ SOC. Those with a greater attachment to local community agreed on a formal variant of citizen participation and mainly focused on electoral behaviors (Brady, 1999; van Deth, 2001). They also emphasized the significance of individual duty and associated citizen participation to development and progress.

Generally, the expectation that high levels of SOC would be associated with a positive representation of citizen participation was supported.

The social representation of medium and low SOC participants conveyed a cooperative and relational image of citizen participation, within which conventional and non-conventional types of political participation, as well as the political and the social form, were blended. Results supported the possibility that average and low attachment to local community does not necessarily result in devaluing citizen participation (Mannarini & Fedi, 2009). On the contrary, they suggested that a weak SOC could be consonant with perspectives that are more comprehensive and inclusive of concerns for both the communities and the broader society and are able to take into account plurality of modes, forms and behaviors.

Ultimately, the study has some limitations that have to be pointed out. First, we adopted a single technique for evoking the social representations of citizen participation but the structural approach suggests the use of diverse instruments. Second, the usage of a sole instrument might limit the interpretation of the results and the comparison between the three groups. Third, we followed the User guide of the EVOC software to set the four areas of the internal structure of the social representations and, as a result, a large quota of less frequent words than expected were included in the central core and, especially, in the periphery of the representations. Fourth, given that sense of community is a dynamic experience embedded in the cultural and historical context, further data are needed to account for the differences that might characterize diverse territories. Nonetheless, we contend that our investigation results opened up a potential route of inquiry on the relationship between low or even negative SOC (Mannarini, Rochira, & Talò, 2014) and citizen participation, supporting the idea that dissatisfaction with the community does not inevitably result in apathy and disengagement, but on the contrary, that it can support a critically constructive view of citizen participation. Future investigations could enlarge the comprehension of the SOC-participation association with regard to multiple SOC, so as to explore to what extent multiple simultaneous belongings can be related to variations in the social representations of citizen participation.
